# The Accuracy of Artificial Intelligence-Based Models Applied to 12-Lead Electrocardiograms for the Diagnosis of Acute Coronary Syndrome: A Systematic Review

**DOI:** 10.1016/j.acepjo.2025.100240

**Published:** 2025-08-22

**Authors:** Aly Fawzy, Aleena Malik, Juan Pablo Diaz-Martinez, Ani Orchanian-Cheff, Sameer Masood

**Affiliations:** 1 Temerty Faculty of Medicine, University of Toronto, Ontario, Canada; 2 Biostatistics Research Unit, University Health Network, Toronto, Ontario, Canada; 3 Library and Information Services, University Health Network, Toronto, Ontario, Canada; 4 Toronto General Hospital Research Institute, University Health Network, Toronto, Ontario, Canada; 5 Division of Emergency Medicine, Department of Medicine, University of Toronto, Toronto, Ontario, Canada

**Keywords:** acute coronary syndrome, AI, ECG, occlusion myocardial infarction, OMI, ST-elevated myocardial infarction, STEMI

## Abstract

**Objectives:**

This systematic review aims to evaluate the diagnostic accuracy of artificial intelligence (AI) algorithms in acute coronary syndrome (ACS) detection using 12-lead electrocardiograms (ECGs).

**Methods:**

Adhering to Preferred Reporting Items for Systematic Reviews guidelines, Ovid MEDLINE, Ovid Embase, Cochrane Central, and Cochrane Database of Systematic Reviews were searched up to June 15, 2023. Eligible studies involved adults with suspected ACS and employed AI for 12-lead ECG interpretation. The primary outcomes were sensitivity and specificity, with secondary outcomes including positive predictive value (PPV), negative predictive value (NPV), and accuracy. Risk of bias was evaluated using Prediction model Risk Of Bias Assessment Tool (PROBAST).

**Results:**

From 2051 records, 24 studies were included. The sensitivity of AI-based diagnosis for ACS among the 24 studies varied from 68% to 98%, and the specificity varied from 41% to 98%. For subgroup analysis of ST-elevated myocardial infarction/occlusion myocardial infarction, sensitivity ranged from 68% to 97% and specificity from 68% to 99%. AI models outperformed clinicians interpreting ECGs retrospectively without knowledge of outcomes in sensitivity (90% of studies) and PPV (100% of studies), whereas clinicians had better NPV (70% of studies). One study compared AI with real-time emergency department physician interpretations. Three studies reported code availability. Thirty-eight percentage of studies showed a high risk of bias, with 50% showing unclear risk, although applicability concerns were minimal.

**Conclusion:**

AI models show high diagnostic accuracy for ACS using 12-lead ECGs, with potential to enhance early diagnosis. However, variability in performance, transparency challenges with limited code availability, a high risk of bias in some studies, and minimal real-time comparisons underscore the necessity for standardized reporting and open-access practices.

## 1 Introduction

### 1.1 Background

Artificial intelligence (AI) has been making significant strides in various domains, including healthcare. Its potential to revolutionize disease diagnosis, patient care, and health system management has been recognized globally.^1^ One of the areas in which AI has shown considerable promise is in the field of cardiology, particularly in the interpretation of 12-lead electrocardiograms (ECGs).^2^ ECG interpretation is a critical aspect in the diagnosis of acute coronary syndrome (ACS), and timely interpretation can impact the morbidity and mortality of patients with ACS.^3^

### 1.2 Importance

AI, particularly machine learning (ML) and deep learning (DL) algorithms, have been applied to ECG interpretation to facilitate ACS diagnosis.^4^ These AI techniques hold promise for enhancing the speed and accuracy of ECG interpretation and improving patient outcomes. Several studies have suggested that AI could potentially identify subtle patterns in ECG tracings that may not be easily recognized by physicians, enabling earlier detection of ACS and prompt initiation of treatment.^4^, ^5^, ^6^, ^7^, ^8^, ^9^, ^10^, ^11^

Despite these promising developments, the evidence base for AI’s effectiveness in this context is fragmented, and its real-world performance is yet to be systematically evaluated. A recent systematic review assessing the application of ML for the diagnosis of ACS using a 12-lead ECG suggested that AI algorithms tend to have better sensitivity in ECG interpretation.^12^ However, the review only included articles that had an AI performance comparator (ie, clinicians, software, or criteria).

### 1.3 Goals of This Investigation

This systematic review will attempt to collate and synthesize the available evidence on the application of AI to ECG for the diagnosis of ACS, assessing its accuracy, reliability, and clinical usefulness. This review will also identify gaps in the current evidence base, providing guidance for future research in this area.

Through this systematic review, we aim to provide a comprehensive evaluation of AI’s role in ACS diagnosis through 12-lead ECG interpretation, potentially informing clinical practice and guiding the adoption of AI algorithms for ECG interpretation in the clinical domain.

## 2 Methods

### 2.1 Study Design and Registration

This systematic review was conducted following the Preferred Reporting Items for Systematic Reviews (PRISMA) guidelines.^13^ The review protocol was registered with the International Prospective Register of Systematic Reviews (PROSPERO) (ID: CRD42023432835). The main objective of this review was to investigate the effectiveness and accuracy of AI techniques in interpreting ECGs for diagnosing ACS.

### 2.2 Search Strategy

A comprehensive search strategy was developed in collaboration with an experienced reference librarian using a combination of database-specific subject headings and text words for the main concepts of AI, ECG, and ACS. Results were limited to adult human studies and the English language. No other limits were applied. We searched the following databases on June 15, 2023: Ovid MEDLINE, Ovid Embase, Cochrane Database of Systematic Reviews (Ovid), and Cochrane Central Register of Controlled Trials (Ovid). See https://doi.org/10.5683/SP3/XLFZCD for full search strategies. The reference lists of retrieved publications were also searched and considered for inclusion.

### 2.3 Selection of Studies

Studies were selected for our review based on a number of inclusion criteria. They were limited to adult patients (≥18 years) who underwent an ECG due to suspicion or diagnosis of ACS in acute care settings including the prehospital setting, emergency departments (EDs), in-patient wards, and intensive care units. The studies also employed AI techniques, as defined by the study authors, for interpreting ECGs in the context of ACS symptoms. Eligible comparators included clinician interpretation (retrospective or real time), commercial ECG software using rule-based criteria, or other AI models, as reported by each study.

The primary outcome of interest was the diagnostic accuracy of ACS, including ACS subtypes (ST-elevated myocardial infarction [STEMI], non-STEMI [NSTEMI], and occlusion MI [OMI]) as determined by AI techniques. We reported the sensitivity and specificity for the primary outcome(s) of diagnostic accuracy of ACS. Secondary outcomes included measures of diagnostic performance including, positive predictive value (PPV), negative predictive value (NPV), accuracy, area under the receiver operator curve (AUROC), and F1 scores. Secondary clinical outcomes included territory of infarct, time to diagnosis, diagnostic accuracy compared with physician decision making, and any reported adverse events.

These outcomes were selected as they represent standard diagnostic performance metrics in AI literature.^1^ Given the heterogeneity in outcome definitions and study methodologies, we did not apply fixed thresholds or conduct a meta-analysis; instead, we used a descriptive synthesis. Additionally, we conducted a subgroup analysis for studies specifically evaluating STEMI/OMI, given its clinical importance.

Studies were excluded if they were case reports, case series, review articles, editorials, or commentaries. Additionally, studies that were still in progress, were not in the English language, did not report on any outcomes or evaluation, did not identify themselves as studying AI or ML, or evaluated AI/ML in stable coronary artery disease were also excluded. Studies that included non-ECG data (ie, clinical or laboratory data) in their AI algorithms or those that used ECG data from large public databases were also excluded from this study. The focus was on studies using ECGs from clinical settings. Furthermore, studies missing basic statistical measures such as the sample size, number of patients with the condition (ie, ACS), sensitivity, or specificity were excluded.

### 2.4 Data Extraction and Synthesis

All records were initially managed through the Zotero reference management software, in which all citations from each database were imported. Two reviewers (AF and AM) independently assessed study titles and abstracts according to the established inclusion criteria. Subsequently, AF and AM examined full texts to assess for eligibility. Any disagreements between the 2 reviewers were resolved through discussion and consultation with a third reviewer (SM). All screening was done using Covidence. A standardized data extraction form was developed and pilot-tested on a sample of included studies (10 studies) to ensure its applicability. Data extraction was led by 1 reviewer (AF) and subsequently confirmed by the second reviewer (AM). Any disagreements in data extraction were resolved through discussion and consultation of the third reviewer (SM).

### 2.5 Data Analysis

Sensitivity and specificity values were extracted from the included studies and used to generate forest plots. Pooled estimates of sensitivity and specificity were calculated to summarize diagnostic performance across studies. To assess bias, the Prediction model Risk Of Bias Assessment Tool (PROBAST) was used to assess the risk of bias in the articles included.^14^ It systematically addressed the following domains: study participants, predictors, outcomes, and statistical analyses. In each of these 4 domains, a scoring scale was used to categorize the risk of bias and applicability concerns into “low,” “high,” or “unclear.” Two reviewers (AF and AM) independently assessed risk of bias using PROBAST. Any discrepancies were resolved by a third reviewer (SM).

## 3 Results

### 3.1 Study Selection

**Figure 1:**
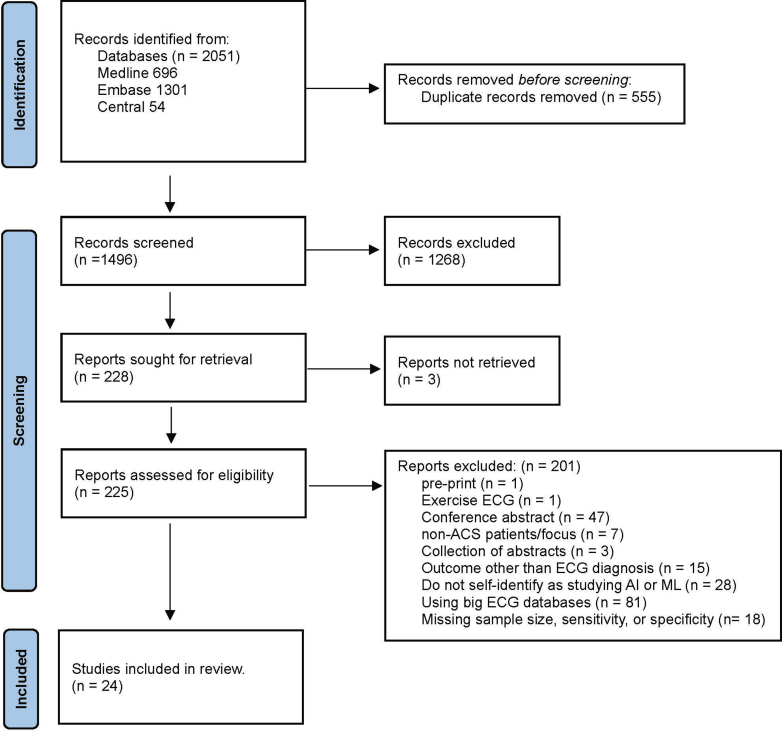
PRISMA flow diagram for study selection process. PRISMA, Preferred Reporting Items for Systematic Reviews.

Our search strategy yielded a total of 2051 articles. After the removal of duplicates, 1496 articles remained for title and abstract screening. After the initial screening, 228 articles were assessed for eligibility through full-text review. For 3 articles, the full text was not retrievable, and these were excluded. After full-text review of the remaining 225 articles, 201 were excluded, with 24 studies meeting the inclusion criteria and were included in the final review (Table S1). The PRISMA flow diagram in Figure 1 provides a detailed overview of the study selection process.

### 3.2 Article Characteristics

The 24 studies included in this review were published over a span of 26 years from 1997 to 2023. These studies originated from a variety of countries, with the majority coming from the United States of America (7 articles), followed by China and South Korean (4 articles each), Sweden (3 articles), Taiwan and Japan (2 articles each), and Germany and Iran (1 article each). The studies varied in terms of their design, specific ACS outcome, and reference standard for ACS diagnosis. Only 3 studies evaluated the AI algorithms’ diagnostic performance in localizing ACS diagnosis. The majority of articles, 75% (18/24), relied on physicians evaluating patient records, which included data from ECGs and laboratory results, to establish the diagnosis of ACS as their ground truth. More specifically, 6 articles mentioned the involvement of 2independent physicians for review, 3 articles reported a review process conducted by 3 independent cardiologists, and 3 articles referenced the discharge summary. A detailed summary of the characteristics of the included studies is provided in Table 1.^15^ Moreover, only 3 out of the 24 studies published their code publicly (Table S2).

**Table 1:** Characteristics of included studies and their outcomes of interest.

First author et al	Year	Country	Study design	ACS outcome of interest	Assess location of infarction	Reference standard for ACS diagnosis	Details of ACS diagnosis by physicians	Non-ACS outcomes reviewed in the study
Al-Zaiti et al	2020	USA	Prospective	MI	No	Physician	2 independent physicians	None
Al-Zaiti et al	2023	USA	Prospective	OMI	No	Physician	2 independent physicians	None
Bouzid et al	2023	USA	Prospective	STEMI and NSTEMI	No	Physician	2 independent physicians	None
Bouzid et al (a)	2021	USA	Prospective	MI	No	Physician	2 independent physicians	None
Bouzid et al (b)	2021	USA	Prospective	MI	Yes	Physician	2 independent physicians	None
Cho et al	2020	South Korea	Retrospective	MI	Yes	CA	NA	None
Choi et al	2023	South Korea	Retrospective	MI	No	CA	NA	None
Choi et al	2022	South Korea	Retrospective	STEMI	No	Physician	7 ED physicians and 3 cardiologists	None
Forberg et al^15^	2009	Sweden	Retrospective	MI	No	Physician	Discharge summary	None
Forberg et al	2012	Sweden	Retrospective	STEMI	No	Physician	2 independent physicians	None
Green et al	2006	Sweden	Retrospective	MI	No	Physician	Discharge summary	None
Hao et al	2020	China	Retrospective	MI	No	Physician	Multiple cardiologists	None
Kaiser et al	1996	Germany	Retrospective	MI	Yes	ECHO or CA	NA	LVH
Kim et al	2022	South Korea	Retrospective	STEMI	No	Physician	Discharge summary	None
Kimura et al	2019	Japan	Retrospective	MI	No	CA	NA	None
Kojuri et al	2015	Iran	Prospective	MI	No	Physician	Unknown	None
Liu et al	2021	Taiwan	Retrospective	STEMI and NSTEMI	No	CA	NA	None
Ouyang et al	1997	Japan	Retrospective	MI	No	Physician	Unknown	None
Polak et al	1997	USA	Retrospective	MI	No	Physician	Unknown	None
Tseng et al	2023	Taiwan	Retrospective	STEMI	No	CA	NA	None
Wang et al	2023	China	Prospective	MI	No	Physician	3 Cardiologists	Arrythmia and LVH
Wu et al (a)	2022	China	Prospective	STEMI	No	Physician	3 Cardiologists	None
Wu et al (b)	2022	China	Prospective	STEMI	No	Physician	3 Cardiologists	None
Xue et al	2001	USA	Prospective	MI	No	Physician	Unknown	None

ACS, acute coronary syndrome; CA, coronary angiography; ECHO, echocardiogram; ED, emergency department; LVH, left ventricular hypertrophy; MI, myocardial infarction; NA, not applicable; NSTEMI, non-ST-elevated myocardial infarction; OMI, occlusion myocardial; STEMI, ST-elevated myocardial infarction.

### 3.3 AI Models and Patient Population

Sample size, patient population characteristics, and the specific AI techniques employed for ECG interpretation in the context of ACS are summarized in Table 2.^15^ The articles used a wide range of AI techniques, with 66.7% (16/24) of the articles employing DL methods (artificial neural networks and convoluted neural networks), 25.0% (6/24) employing traditional ML methods (logistic regression, gradient boosting machine, random forest, long short-term memory, and least absolute shrinkage and selection operator), and 3% (2/24) employing hybrid methods (adaptive logic computing network and fusions).

**Table 2:** Patient demographics of included studies and AI models used.

First author et al	Year	AI model used	Sample size, n	Mean age, y	Females, %	Patients with ACS, %(n)	No. of ECGs, n	ECGs with ACS, %(n)
Al-Zaiti et al	2020	Fusion: LR + GBM + ANN	1224	59	42	16.8 (206)	1224	16.8 (206)
Al-Zaiti et al	2023	RF	7313	59	47	14.9 (1087)	7313	14.9 (1087)
Bouzid et al	2023	RF	2122	59	53	13.6 (288)	NR	NR
Bouzid et al (a)	2021	LR	1244	59	49	16.6 (206)	1244	16.6 (206)
Bouzid et al (b)	2021	RF	2400	59	47	15.8 (380)	2400	15.8 (380)
Cho et al	2020	DL	1768	62	62	12.7 (225)	1768	12.7 (225)
Choi et al	2023	DL	10,160	62	20	16.6 (1689)	10,160	16.6 (1689)
Choi et al	2022	CNN	187	62	25	51.3 (96)	NR	NR
Forberg et al	2009	ANN	861		40	40.0 (344)	861	NR
Forberg et al^15^	2012	ANN	560	70	45	17.5 (98)	560	17.5 (98)
Green et al	2006	ANN	634	65	43	20.5 (130)	NR	
Hao et al	2020	CNN	957	NR	NR	50.5 (483)	957	50.5 (483)
Kaiser et al	1996	Rule-based learning	605			70.9 (429)	605	70.9 (429)
Kim et al	2022	CNN	80	65	21	67.5 (54)	80	67.5 (54)
Kimura et al	2019	Neural network - bidirectional LSTM	792	NR	NR	14.3 (113)	NR	NR
Kojuri et al	2015	ANN multilayer perceptron	935	NR	56	8.8 (82)	NR	NR
Liu et al	2021	DL	77,799	NR	NR	1.3 (1024)	142084	1.3 (1748)
Ouyang et al	1997	ANN	132	50	NR	25.0 (33)	NR	NR
Polak et al	1997	Adaptive logic computing network	1367	NR	54	3.7 (50)	NR	NR
Tseng et al	2023	CNN	384	NR		34.4 (132)	NR	NR
Wang et al	2023	CNN	3392	39	3.66	3.5 (118)	NR	NR
Wu et al (a)	2022	CNN-LTSM	883	NR	33	35.7 (315)	NR	NR
Wu et al (b)	2022	LASSO	820	NR	34	7.2 (59)	259	NR
Xue et al	2001	Neural network	2308	NR	NR	58.0 (1339)	NR	NR

ACS, acute coronary syndrome; AI, artificial intelligence; ANN, artificial neural network; CNN, convolutional neural network; DL, deep learning; ECG, electrocardiogram; GBM, gradient boosting machine; LASSO, least absolute shrinkage and selection operator; LR, logistic regression; LSTM, long short-term memory; NR, not reported; RF, random forest.

**Figure 2:**
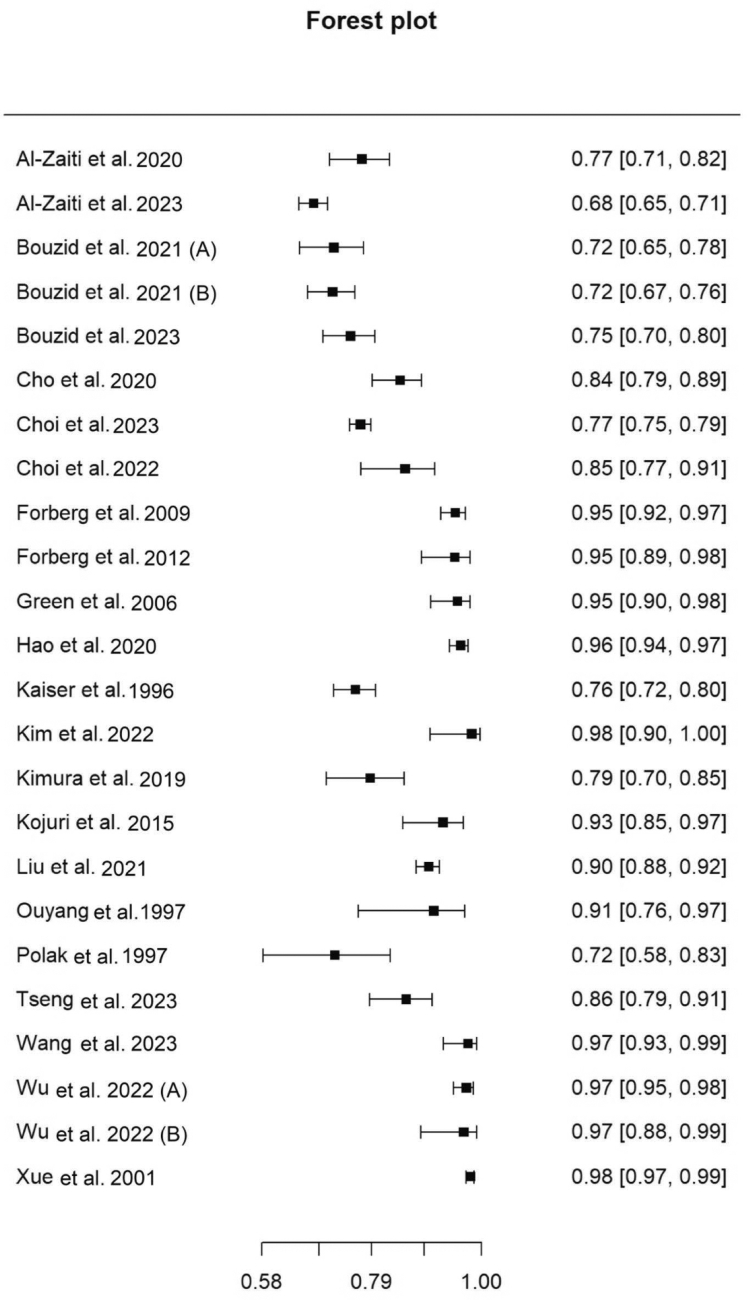
Forest plot of sensitivity of included studies. Pooled sensitivity, 88.9% (95% CI, 84.1%-92.4%).

**Figure 3:**
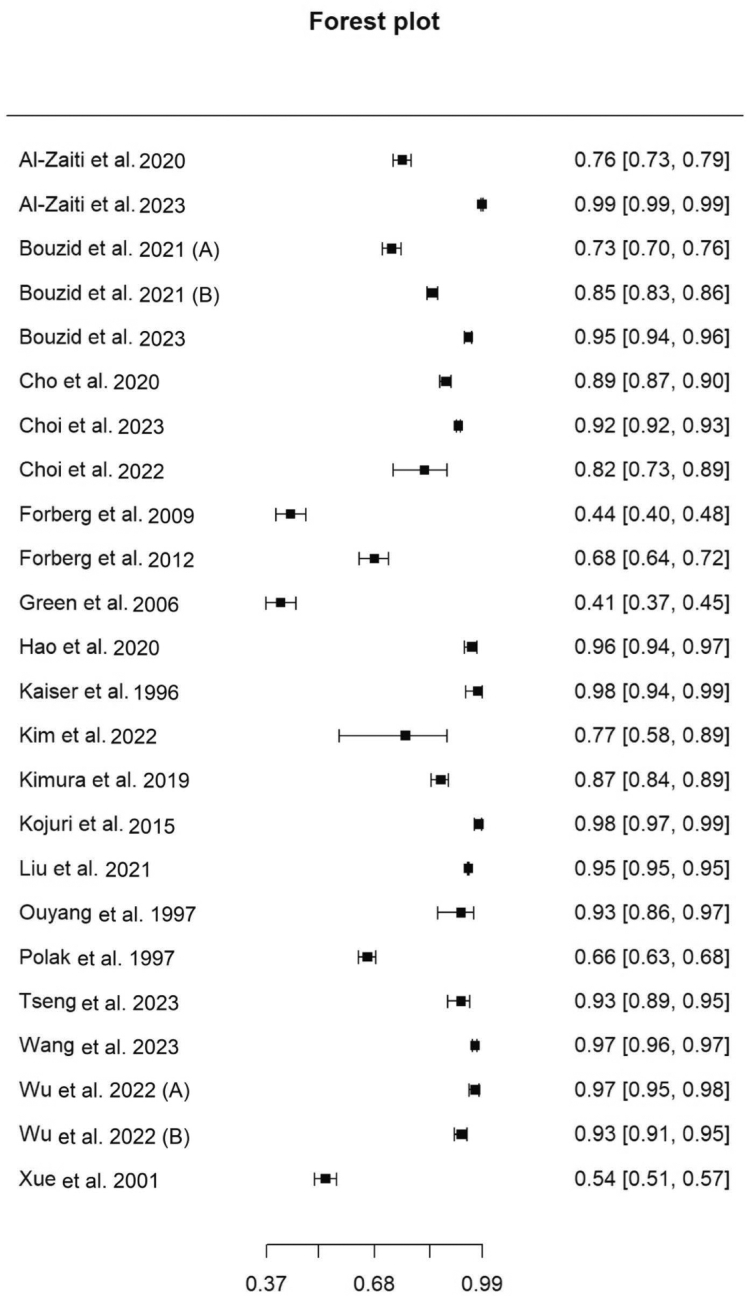
Forest plot of specificity of included studies. Pooled specificity, 88.7% (95% CI, 82.1%-93.1%).

Table 3 summarizes the diagnostic performance metrics for the 24 studies. The sensitivity of AI-based diagnosis for ACS among the 24 studies varied from 68% to 98%, and the specificity varied from 41% to 98% (Figs 2 and 3, respectively).

**Table 3:** Diagnostic performance metrics of artificial intelligence models for acute coronary syndrome diagnosis using electrocardiogram interpretation.

First author et al	Year	Sensitivity, %	Specificity, %	AUROC, %	F1 score, %	Accuracy, %	PPV, %	NPV, %
Al-Zaiti et al	2020	77	76	82	NR	NR	43	NR
Al-Zaiti et al	2023	68	98.9	79	NR	NR	82.5	92.5
Bouzid et al	2023	75	95	83	NR	NR	NR	NR
Bouzid et al (a)	2021	72	73	79	NR	NR	38	92
Bouzid et al (b)	2021	71.71	84.73	85	NR	NR	36.13	96.13
Cho et al	2020	84.4	88.5	90.1	NR	NR	51.8	97.5
Choi et al	2023	76.9	92.1	92.3	75.8	88.5	NR	NR
Choi et al	2022	85.4	82.4	91.9	NR	NR	83.7	84.3
Forberg et al^15^	2009	95	44	86	NR	NR	53	94
Forberg et al	2012	95	68	93	NR	NR	18	99
Green et al	2006	95	41.1	80.2	NR	NR	29.5	97.2
Hao et al	2020	96	96	NR	94	95	NR	NR
Kaiser et al	1996	76	98	NR	NR	NR	99	NR
Kim et al	2022	98	77	95	NR	NR	90	95
Kimura et al	2019	79	87	88	NR	83	NR	NR
Kojuri et al	2015	93	98	NR	NR	96	99	82
Liu et al	2021	90	95	98	NR	NR	NR	NR
Ouyang et al	1997	90.2	93.3	NR	NR	91.8	NR	NR
Polak et al	1997	72	66	NR	NR	67	NR	NR
Tseng et al	2023	85.9	92.9	NR	NR	83.7	NR	NR
Wang et al	2023	97.1	96.88	NR	NR	97.2	NR	NR
Wu et al (a)	2022	97	97	100	98	98	99	99
Wu et al (b)	2022	97	93	98	NR	95	93	97
Xue et al	2001	98	54	NR	NR	NR	NR	NR

AUROC, area under receiver operating curve; NPV, negative predictive value; NR, not reported; PPV, positive predictive value.

### 3.4 Subgroup Analysis

Among the 24 studies reviewed, 7 specifically evaluated AI’s diagnostic performance for STEMI/OMI. The sensitivity of AI-based diagnosis for STEMI/OMI among the 7 studies varied from 68% to 97%, and the specificity varied from 68% to 99% (Figs S1 and S2, respectively).

### 3.5 Comparative Analysis of Diagnostic Performance

Out of the 24 articles, 75.0% (n = 18/24) compared their AI algorithm’s diagnostic performance to another modality. The comparative diagnostic performance is summarized in Table 4.^15^ The most common comparison was against the performance of a group of expert clinicians in diagnosing ACS events (MI, STEMI, and OMI) based on ECGs (33%, 8/24). These evaluations were conducted retrospectively and blinded to clinical outcomes to minimize bias. Only 1 study (Forberg et al 2012) compared their AI model with the real-time interpretation of ECGs by coronary care unit physicians.^15^ One study (Kim et al 2022), in addition to expert clinician performance, also reported real-time performance of emergency medicine physicians.^16^ Twenty-five percent of articles (6/24) compared their AI performance with commercial non-ML software. These tools rely on predefined rules or criteria, such as the HEART score^17^ and classical ECG criteria,^15^ rather than advanced ML techniques. Three articles (12.5%) compared their AI model’s diagnostic performance with that of other AI articles.

**Table 4:** Diagnostic performance metrics of clinicians and non-AI commercial software for ACS diagnosis using electrocardiogram interpretation.

First author et al	Year	ACS event assessed	AI comparator	Expert clinicians					Software				
				Sensitivity, %	Specificity, %	AUROC, %	PPV, %	NPV, %	Sensitivity, %	Specificity, %	AUROC, %	PPV, %	NPV, %
Al-Zaiti et al^a^	2020	Any ACS event	Clinicians + software + criteria	40	94	67	63	87	25	98	62	79	85
Al-Zaiti et al	2023	OMI	Clinicians + software	NR	NR	72	NR	NR	NR	NR	68	NR	NR
Bouzid et al	2023	Any ACS event	Clinicians	35.7	85.8	62	27.8	89.7	NR	NR	NR	NR	NR
Bouzid et al (a)	2021	Any ACS event	Clinicians + software	40	94	NR	63	88	25	98	NR	79	85
Bouzid et al (b)	2021	Any ACS event	Software	NR	NR	NR	NR	NR	31.21	95.61	NR	50.53	92.66
Cho et al^b^	2020	MI	Software	NR	NR	NR	NR	NR	81.9	81.4	NR	15.8	99.1
Choi et al	2023	MI	Software	NR	NR	NR	NR	NR	72.8	62.2	72.8	NR	NR
Choi et al	2022	STEMI	Clinicians	87.5	58.2	85.6	68.8	81.6	NR	NR	NR	NR	NR
Forberg et al^15^^c^	2009	Any ACS event	Clinician + criteria	82	63	78	39	91	NR	NR	NR	NR	NR
Forberg et al^e^	2012	STEMI	Clinicians	74	98	NR	76	98	NR	NR	NR	NR	NR
Green et al	2006	NA	Other AI models	NR	NR	NR	NR	NR	NR	NR	NR	NR	NR
Hao et al	2020	NA	None	NR	NR	NR	NR	NR	NR	NR	NR	NR	NR
Kaiser et al	1996	NA	None	NR	NR	NR	NR	NR	NR	NR	NR	NR	NR
Kim et al^f^	2022	STEMI	Clinicians	94	58	76	82	83	NR	NR	NR	NR	NR
Kimura et al	2019	NA	None	NR	NR	NR	NR	NR	NR	NR	NR	NR	NR
Kojuri et al	2015	NA	None	NR	NR	NR	NR	NR	NR	NR	NR	NR	NR
Liu et al	2021	STEMI	Clinicians + software	NR	NR	NR	NR	NR	NR	NR	NR	NR	NR
Ouyang et al	1997	NA	None	NR	NR	NR	NR	NR	NR	NR	NR	NR	NR
Polak et al	1997	MI	Software	NR	NR	NR	NR	NR	76	54	NR	NR	NR
Tseng et al	2023	NA	Other AI models	NR	NR	NR	NR	NR	NR	NR	NR	NR	NR
Wang et al	2023	NA	Other AI models	NR	NR	NR	NR	NR	NR	NR	NR	NR	NR
Wu et al (a)^d^	2022	STEMI	Clinicians	92	92	92	92	92	NR	NR	NR	NR	NR
Wu et al (b)	2022	STEMI	Clinicians	72	93	83	75	94	NR	NR	NR	NR	NR
Xue et al	2001	NA	None	NR	NR	NR	NR	NR	NR	NR	NR	NR	NR

ACS, acute coronary syndrome; AI, artificial intelligence; AUROC, area under receiver operating curve; MI, myocardial infarction; NA, not applicable; NPV, negative predictive value; NR, not reported; OMI, occlusion myocardial infarction; PPV, positive predictive value; STEMI, ST-elevated myocardial infarction.

a Criteria AUROC: 84.

b Software F1 and accuracy scores: 67 for both.

c Criteria AUROC, sensitivity, specificity, PPV, and NPV; 76, 75, 36, 39, and 91, respectively.

d Clinician F1 and accuracy scores: 92 for both.

e Real-time interpretation by coronary care unit physicians.

f Comparison to real-time interpretation by emergency medicine physicians; sensitivity, specificity, PPV, and NPV; 57, 85, 89, and 49, respectively.

AI models were more sensitive (90% of studies, n = 9/10) and had better PPV (100% of studies, n = 10/10) compared with clinicians. However, specificity varied, with only 50% of the studies (n = 5/10) showing that AI models were more specific compared with clinicians, whereas the remaining studies showed comparable or superior clinician specificity. Clinicians had better NPV (70% of studies, n = 7/10) compared with AI models. Additionally, AI models had better AUROC compared with clinicians in all 8 studies that reported on that metric.

In the comparison of AI models to commercial non-ML software across 7 studies, AI models consistently demonstrated higher sensitivity in diagnosing ACS (86% of studies, n = 6/7). Specificity results were mixed, with 57% of studies (n = 4/7) reporting superior specificity of AI models. AI models had better PPV compared with software in 83% of studies (n = 5/6). However, software had better NPV compared with AI models in 60% of studies (n = 3/5).

### 3.6 Risk of Bias Assessment

Table S3 delineates the PROBAST evaluation of risk of bias and applicability considerations for the articles included in the systematic review. The analysis revealed that the majority of studies demonstrated an unclear risk of bias in at least 1 domain—most notably within predictors, outcome, and analysis. The overall risk of bias was pronounced, with a high concern identified in 38% of the studies (n = 9/24) and an unclear concern observed in half of the studies (Fig S3). The participant domain, along with the analysis domain—specifically regarding the adequacy of participant numbers and the handling of missing data—were recurrent sources of high risk. The high risk of bias was not apparent in the outcome and predictors domain, with none of the studies receiving a rating of high risk, and only 17% (n = 4/24) and 21% (n = 5/24), respectively, marked as unclear. This suggests that biases related to outcome determination and predictor selection were less prevalent. Applicability concerns were minimal, reflecting the studies’ alignment with the populations, predictors, and outcomes detailed in the review question; only a small fraction exhibited high (13%, n = 3/24) or unclear (8%, n = 2/24) overall applicability risk (Fig S4).

## 4 Limitations

Our systematic review is not without limitations. We excluded studies that used ECGs from databases, which several articles have used to develop AI models. This approach was taken to focus on real-world data at the expense of excluding some of these AI models. Moreover, we excluded articles with missing sensitivities and specificities. The evaluation of a model’s performance cannot be fully encapsulated merely by its sensitivity and specificity, as some models offer the ability to adjust the threshold, thus balancing the trade-off between sensitivity and specificity.

Additionally, our literature search was conducted in June 2023. Given the rapid pace of advancement in the field of AI, particularly in ECG interpretation, it is possible that relevant studies published after this date were not captured. As such, our findings may not fully reflect the most recent developments in AI-based diagnostic tools for ACS.

In the comparative analysis between AI performance and clinician performance, the majority of articles used a group of physicians that retrospectively annotated the ECGs. This might have overestimated the diagnostic performance of clinicians as compared with AI and may not reflect real-world performance. Only 1 study compared their model with real-time ED physician interpretation.^15^ Future research should explore this comparison to provide a more comprehensive understanding of AI’s practical utility in dynamic clinical environments.

In assessing the clinical significance of AI models in the diagnosis of ACS using ECGs, our analysis revealed considerable heterogeneity in outcomes, underscoring an imperative limitation in directly comparing various AI approaches. This variability stems from a multitude of factors, including the distinct design methodologies of AI models and the diverse patient populations studied. Variability in reference standards for diagnosing STEMI or OMI across studies, including differences in criteria such as complete occlusion, thrombosis in myocardial infarction flow grades, and the identification of culprit lesions, may have also influenced the consistency and comparability of reported diagnostic performance metrics.

Transparency in AI development remains a significant challenge, particularly with the prevalence of proprietary licensing and limited code availability. As noted in Table S2, although several models showed superior performance in the detection of ACS, most studies did not disclose their algorithms, which could impede validation and integration into clinical practice. This introduces an additional layer of opacity, restricting the reproducibility and validation of the reported AI algorithms. Overcoming this barrier will require a commitment to open science and standardization of reporting protocols. Additionally, although AI tools for ECG interpretation may initially be limited to cardiologists, developing accessible systems for ED settings can help frontline physicians leverage AI for faster, more accurate decision making, enhancing workflows without disruption.

Although this study did not include a cost analysis of AI technologies, the economic implications are critical for their widespread adoption in clinical settings. Adopting AI tools requires addressing the learning curve through Continuing Medical Education programs that teach clinicians to understand predictions, limitations, and effective integration into patient care. Future studies should evaluate the cost effectiveness of these technologies, including an analysis of particular brands and their performance, to better understand their potential for integration into healthcare systems.

Lastly, there is also a need to test if these AI models led to a change in outcome and any associated adverse events, including time to diagnosis. It is assumed that early and timely diagnosis of ACS would lead to better outcomes; however, this has to be explored and quantified. Similarly, the impact of AI models on identifying the territory of infarct was also not adequately addressed in this review, as very few studies addressed this outcome. Territory of infarct is a critical aspect of ACS diagnosis and can have implications on emergent management.

## 5 Discussion

### 5.1 Our Study

Our study is the largest study to examine the application of ML algorithms, not limited to DL, for diagnosing ACS using ECGs in real-world clinical settings. Our review incorporates studies from multicenter populations presenting with suspected ACS in emergency care settings, thereby enhancing external validity and generalizability. We chose to include studies that also compare the diagnostic accuracy of their algorithms with other published articles and not only limit it to articles with direct AI-to-clinician comparisons.

### 5.2 AI in 12-Lead ECG Interpretation to Diagnosis ACS

Our systematic review included 24 articles, which collectively demonstrated that AI algorithms exhibit high diagnostic performance in detecting ACS using ECGs in acute care settings. Across the studies, reported sensitivities ranged from 68% to 98% and specificities from 41% to 98%, highlighting the variability in performance across different AI models and study designs. In comparison, the sensitivity and specificity of manual acute MI diagnosis have been reported as 91% and 51%, respectively.^18^ This shows the potential for AI in enhancing diagnostic speed and potentially reducing human error when it comes to ACS. Similar work has been conducted in which AI ECG analyses were evaluated for their prediction of structural cardiac pathologies, including, left ventricular systolic dysfunction, myocardial hypertrophy, heart failure, silent atrial fibrillation, hypertrophic cardiomyopathy, and bundle branch blocks.^4^^,^^19^, ^20^, ^21^, ^22^, ^23^ Two recent studies by Herman et al,^24^^,^^25^ published after the conclusion of our search, examined 2 large patient populations and demonstrated that an AI-powered ECG system outperformed current computerized interpretations in diagnostic accuracy across various cardiac conditions. These studies, achieving high F1 scores, align with our conclusions, reinforcing the potential of AI-powered ECG systems as reliable clinical tools.

### 5.3 STEMI/OMI

Subgroup analysis revealed that AI’s diagnostic performance for STEMI/OMI showed higher sensitivity and specificity, suggesting that AI might be more effective in this context. However, this improvement does not fully explain the heterogeneity observed in the overall results, indicating that other factors contribute to the variability across studies. Although AI appears better suited for diagnosing STEMI/OMI, further research is needed to understand the sources of heterogeneity and to validate these findings across broader clinical scenarios. Future advancements in AI algorithms should prioritize this subset, as timely detection of OMIs is critical to optimizing patient outcomes in acute care settings.

### 5.4 Comparative Analysis: AI vs Clinician and Software Performance

Our review showed that for the diagnosis of ACS, AI models had higher sensitivity than clinicians while maintaining similar specificity. Similarly, AI models showed superior PPV; however, those gains were balanced by decreases in NPV. The gains in sensitivity and PPV show AI’s potential to be used for ruling out ACS in acute care settings. Furthermore, the higher AUROC values seen in ECG-based AI models, positions it well to be used as a discriminatory tool for ACS.

When AI models were compared with commercial software, the results were similarly encouraging for AI, with gains in sensitivity, and PPV while maintaining specificity and NPV. Overall, however, NPV and AUROC results were less consistently reported for software comparisons, suggesting that more research is needed to fully understand the performance of AI vs non-ML commercial software. These findings indicate that AI holds considerable promise for enhancing the diagnosis of ACS. It may improve early detection and reduce false negatives, which are critical in acute settings. Yet, the importance of clinician expertise remains, particularly in avoiding false positives and providing comprehensive patient care that considers the nuances AI might miss. These aspects highlight crucial considerations for integrating AI into clinical practice, especially regarding how AI could augment clinician judgment.

### 5.5 Risk of Bias Assessment

The PROBAST evaluation conducted in this systematic review serves to highlight key areas within the current body of literature on AI algorithms for ACS diagnosis via ECG that require careful consideration. Notably, the prevalence of unclear risk of bias across several domains raises concerns about the methodologic transparency in these studies. Specifically, the high risk of bias in 38% of the included studies underscores a critical need for more rigorous methodologic design and reporting, particularly in participant recruitment and the statistical handling of data. Although none of the studies showed a high risk of bias for outcome and predictors, a significant proportion exhibited unclear risk (17% for outcomes and 21% for predictors), indicating that there are still gaps in how these aspects are being reported. Without clear and comprehensive reporting of how outcomes and predictors are determined, the potential for bias cannot be adequately assessed, which casts doubt on the reliability and applicability of the findings.

Furthermore, the minimal concerns regarding applicability are promising, suggesting that the studies are relevant to the populations, predictors, and outcomes of interest. This relevance is paramount for the translation of research findings into clinical practice. Nevertheless, the presence of any high risk of applicability (13%) cannot be overlooked as it may signal a disconnect between the research settings and real-world clinical environments.

The identified areas of high and unclear risk in our PROBAST analysis reflect a broader issue in the field of AI research in healthcare—namely the necessity for a standardized approach to the reporting and validation of AI models. As AI continues to advance, it is imperative that these models are developed and reported with a level of rigor that instills confidence in their predictive capabilities and their potential for integration into clinical decision-making processes.

### 5.6 Clinical Significance

The broader implications of these findings suggest that AI could play a critical role in settings in which immediate expert interpretation of ECG is not available in real time, thus supporting timely clinical decision making. As we consider the integration of AI into clinical workflows, it is imperative to recognize the balance required between AI’s high sensitivity and the need to mitigate potential false positives, which could be informed by the clinician’s expertise and consideration of the patient’s overall clinical picture. AI’s role in ECG interpretation is not intended to replace the clinical judgment of ED physicians but rather to complement their expertise. By providing high sensitivity and rapid assessments, AI can enhance decision making while allowing physicians to focus on holistic patient care. Maintaining this balance is essential for maximizing AI’s benefits without diminishing the critical contributions of human clinicians. Although our study elucidates the strengths of AI in ACS diagnosis, it also identifies critical areas for future investigation, particularly concerning the integration of AI in the clinical decision-making process and the consequent impact on patient outcomes. It is this potential to contribute to earlier and more accurate ACS diagnoses, ultimately leading to timely and effective patient management, that underpins the clinical significance of our systematic review.

Our systematic review suggests that AI-based algorithms perform well in the diagnosis of ACS using ECGs. Notably, AI has demonstrated superior performance to clinicians and standard software in many instances, albeit with considerable variability in specificity and NPV. Such variability calls for judicious use of AI, complementing rather than supplanting clinician judgment. The heterogeneity of outcomes limits direct comparison of the various AI techniques, emphasizing the need for standardized protocols, publishing of ML codes publicly, and clear interpretability in AI solutions. The risk of bias assessment underscored the importance of addressing methodologic and reporting inconsistencies in AI research. Moving forward, further research is crucial to fine-tune AI tools for consistent and transparent clinical application, ultimately improving patient outcomes. By improving these areas, future studies can enhance the strength of evidence for AI applications in diagnosing ACS and ultimately aid in the advancement of precision medicine.

## Author Contributions

All authors have made substantial contributions to this study. AF was the first reviewer, contributing to the study design and protocol creation. AM was the second reviewer. Both AF and AM were involved in full-text screening, with AF taking the lead in data extraction. AO and JM were responsible for the development and execution of the search strategy. SM conceptualized the study, provided supervision throughout the review process, and resolved screening conflicts. All authors were involved the preparation and review of the manuscript.

## Funding and Support

By *JACEP* *Open* policy, all authors are required to disclose any and all commercial, financial, and other relationships in any way related to the subject of this article as per ICMJE conflict of interest guidelines (see www.icmje.org). The authors have stated that no such relationships exist.

## Conflict of Interest

All authors have affirmed they have no conflicts of interest to declare.
