# Supplementary material for: The Accuracy of Artificial Intelligence-Based Models Applied to 12-Lead Electrocardiograms for the Diagnosis of Acute Coronary Syndrome: A Systematic Review
Source: J Am Coll Emerg Physicians Open. 2025 Aug 22;6(5):100240. doi: 10.1016/j.acepjo.2025.100240 (PMC12529686; doi:10.1016/j.acepjo.2025.100240)
Supplement: Supplementary Figures 1-4 and Supplementary Tables 1-3 [file mmc1.docx]

**Supplementary Material**

**Table S1:** Papers included.

**Table S2:** Code availability

**Table S3:** PROBAST analysis for the evaluation of risk of bias and applicability in studies.

**Figure S1:** Forest plot of sensitivity of studies evaluating ST elevation myocardial infarction (STEMI) or occlusion myocardial infarction (OMI). Pooled sensitivity 92.5% (95% CI: 84.1%-96.6%).

**Figure S2:** Forest plot of specificity of studies evaluating ST elevation myocardial infarction (STEMI) or occlusion myocardial infarction (OMI). Pooled sensitivity 92.0% (95% CI: 81.0%-97.0%).

**Figure S3:** Risk of bias assessment using PROBAST analysis. Traffic plot (A) and summary (B).

**Figure S4:** Applicability assessment using PROBAST analysis. Traffic plot (A) and summary (B).

**Table S1:** Papers included.

| First Author et al. | Year | Title | DOI |
| --- | --- | --- | --- |
| Al-Zaiti et al. | 2020 | Machine learning-based prediction of acute coronary syndrome using only the pre-hospital 12-lead electrocardiogram | <https://doi.org/10.1038/s41467-020-17804-2> |
| Al-Zaiti et al. | 2023 | Machine learning for ECG diagnosis and risk stratification of occlusion myocardial infarction | <https://doi.org/10.1038/s41591-023-02396-3> |
| Bouzid et al. | 2023 | Incorporation of Serial 12-Lead Electrocardiogram With Machine Learning to Augment the Out-of- Hospital Diagnosis of Non-ST Elevation Acute Coronary Syndrome | <https://doi.org/10.1016/j.annemergmed.2022.08.005> |
| Bouzid et al. (A) | 2021 | In Search of an Optimal Subset of ECG Features to Augment the Diagnosis of Acute Coronary Syndrome at the Emergency Department | <https://doi.org/10.1161/JAHA.120.017871> |
| Bouzid et al. (B) | 2021 | Novel ECG features and machine learning to optimize culprit lesion detection in patients with suspected acute coronary syndrome | <https://doi.org/10.1016/j.jelectrocard.2021.07.012> |
| Cho et al. | 2020 | Artificial intelligence algorithm for detecting myocardial infarction using six‐lead electrocardiography | <https://doi.org/10.1038/s41598-020-77599-6> |
| Choi et al. | 2023 | Electrocardiogram-based deep learning algorithm for the screening of obstructive coronary artery disease | <https://doi.org/10.1186/s12872-023-03326-4> |
| Choi et al. | 2022 | Artificial intelligence versus physicians on interpretation of printed ECG images: Diagnostic performance of ST-elevation myocardial infarction on electrocardiography | <https://doi.org/10.1016/j.ijcard.2022.06.012> |
| Forberg et al. | 2009 | In search of the best method to predict acute coronary syndrome using only the electrocardiogram from the emergency department | <https://doi.org/10.1016/j.jelectrocard.2008.07.010> |
| Forberg et al. | 2012 | An artificial neural network to safely reduce the number of ambulance ECGs transmitted for physician assessment in a system with prehospital detection of ST elevation myocardial infarction | <https://doi.org/10.1186/1757-7241-20-8> |
| Green et al. | 2006 | Comparison between neural networks and multiple logistic regression to predict acute coronary syndrome in the emergency room | <https://doi.org/10.1016/j.artmed.2006.07.006> |
| Hao et al. | 2020 | Multi-branch fusion network for Myocardial infarction screening from 12-lead ECG images | <https://doi.org/10.1016/j.cmpb.2019.105286> |
| Kaiser et al. | 1996 | A Practical Example of Using Artificial Intelligence to Improve Computer-based Detection of Myocardial Infarction and Left Ventricular Hypertrophy in the 12-Lead ECG | <https://doi.org/10.1016/s0022-0736(96)80004-5> |
| Kim et al. | 2022 | A Retrospective Clinical Evaluation of an Artificial Intelligence Screening Method for Early Detection of STEMI in the Emergency Department | <https://doi.org/10.3346/jkms.2022.37.e81> |
| Kimura et al. | 2019 | Artificial intelligence to predict needs for urgent revascularization from 12-leads electrocardiography in emergency patients | <https://doi.org/10.1371/> |
| Kojuri et al. | 2015 | Prediction of acute myocardial infarction with artificial neural networks in patients with nondiagnostic electrocardiogram | <http://dx.doi.org/10.5530/jcdr.2015.2.2> |
| Liu et al. | 2021 | A deep learning algorithm for detecting acute myocardial infarction | <https://doi.org/10.4244/eij-d-20-01155> |
| Ouyang et al. | 1997 | Use of an artificial neural network to analyse an ECG with QS complex in V 1-2 leads | <https://doi.org/10.1007/BF02525541> |
| Polak et al. | 1997 | Using automated analysis of the resting twelve- lead ECG to identify patients at risk of developing transient myocardial ischaemia - an application of an adaptive logic network | <https://doi.org/10.1088/0967-3334/18/4/005> |
| Tseng et al. | 2023 | Identification of Coronary Culprit Lesion in ST Elevation Myocardial Infarction by Using Deep Learning | <https://doi.org/10.1109%2FJTEHM.2022.3227204> |
| Wang et al. | 2023 | Convolutional Neural Network-Based ECG-Assisted Diagnosis for Coal Workers | <https://doi.org/10.3390/ijerph20010009> |
| Wu et al. (A) | 2022 | Deep Learning Networks Accurately Detect ST-Segment Elevation Myocardial Infarction and Culprit Vessel | <https://doi.org/10.3389/fcvm.2022.797207> |
| Wu et al. (B) | 2022 | LASSO Regression-Based Diagnosis of Acute ST-Segment Elevation Myocardial Infarction (STEMI) on Electrocardiogram (ECG) | <https://doi.org/10.3390/jcm11185408> |
| Xue et al. | 2001 | A New Method to Incorporate Age and Gender Into the Criteria for the Detection of Acute Inferior Myocardial Infarction | <https://doi.org/10.1054/jelc.2001.28904> |

**Table S2:** Code availability

| First Author et al. | Year | Code published | Details |
| --- | --- | --- | --- |
| Al-Zaiti et al. | 2020 | No | Available from the corresponding author upon request. Copyrighted by University of Pittsburgh |
| Al-Zaiti et al. | 2023 | **Yes** | Available on GitHub |
| Bouzid et al. | 2023 | No | Authors do not comment on code availability |
| Bouzid et al. (A) | 2021 | No | Authors do not comment on code availability. Code has a patent |
| Bouzid et al. (B) | 2021 | No | Authors do not comment on code availability. Code has a patent |
| Cho et al. | 2020 | No | Authors do not comment on code availability |
| Choi et al. | 2023 | No | Authors do not comment on code availability |
| Choi et al. | 2022 | No | Authors do not comment on code availability |
| Forberg et al. | 2009 | No | Authors do not comment on code availability |
| Forberg et al. | 2012 | No | Authors do not comment on code availability |
| Green et al. | 2006 | No | Authors do not comment on code availability |
| Hao et al. | 2020 | No | Authors do not comment on code availability |
| Kaiser et al. | 1996 | No | Authors do not comment on code availability |
| Kim et al. | 2022 | No | Authors do not comment on code availability |
| Kimura et al. | 2019 | **Yes** | Published as supplementary material |
| Kojuri et al. | 2015 | No | Authors do not comment on code availability |
| Liu et al. | 2021 | No | Authors do not comment on code availability |
| Ouyang et al. | 1997 | No | Authors do not comment on code availability |
| Polak et al. | 1997 | No | Authors do not comment on code availability |
| Tseng et al. | 2023 | No | Authors do not comment on code availability |
| Wang et al. | 2023 | No | Authors do not comment on code availability |
| Wu et al. (A) | 2022 | **Yes** | Available on GitHub |
| Wu et al. (B) | 2022 | No | Available from the corresponding author upon reasonable request. |
| Xue et al. | 2001 | No | Authors do not comment on code availability |

**Table S3:** PROBAST analysis for the evaluation of risk of bias and applicability in studies.

|  |  | Risk of Bias | | | | | Applicability | | | | Type of prediction study |
| --- | --- | --- | --- | --- | --- | --- | --- | --- | --- | --- | --- |
| First Author et al. | **Year** | Participant | Predictors | Outcome | Analysis | Overall | Participant | Predictors | Outcome | Overall |  |
| Al-Zaiti et al. | 2020 | **-** | - | - | ? | ? | - | - | - | - | D & V |
| Al-Zaiti et al. | 2023 | **-** | ? | ? | ? | ? | - | - | - | - | D & V |
| Bouzid et al. | 2023 | **-** | ? | ? | - | ? | ? | + | ? | ? | D & V |
| Bouzid et al. (A) | 2021 | **-** | - | - | - | - | - | - | - | - | D & V |
| Bouzid et al. (B) | 2021 | **-** | ? | - | - | ? | - | - | - | - | D & V |
| Cho et al. | 2020 | **-** | ? | ? | + | + | - | - | - | - | D & V |
| Choi et al. | 2023 | **-** | - | - | ? | ? | - | - | - | - | D & V |
| Choi et al. | 2022 | **-** | - | - | ? | ? | - | - | - | - | D & V |
| Forberg et al. | 2009 | **+** | ? | - | - | + | - | - | - | - | D & V |
| Forberg et al. | 2012 | **-** | - | - | ? | ? | - | - | - | - | D & V |
| Green et al. | 2006 | **-** | - | - | + | + | - | - | - | - | D & V |
| Hao et al. | 2020 | **-** | - | ? | + | + | - | - | - | - | D |
| Kaiser et al. | 1996 | **-** | - | - | - | - | - | - | ? | ? | D & V |
| Kim et al. | 2022 | **?** | - | - | ? | ? | + | - | - | + | D & V |
| Kimura et al. | 2019 | **?** | - | - | ? | ? | - | - | - | - | D & V |
| Kojuri et al. | 2015 | **+** | - | - | ? | + | + | - | - | + | D & V |
| Liu et al. | 2021 | **+** | - | - | ? | + | + | - | - | + | D & V |
| Ouyang et al. | 1997 | **+** | - | - | ? | + | - | - | - | - | D & V |
| Polak et al. | 1997 | **-** | - | - | ? | ? | - | - | - | - | D & V |
| Tseng et al. | 2023 | **-** | - | - | ? | ? | - | - | - | - | D & V |
| Wang et al. | 2023 | **-** | - | - | + | + | - | - | - | - | D & V |
| Wu et al. (A) | 2022 | **-** | - | - | + | + | - | - | - | - | D & V |
| Wu et al. (B) | 2022 | **-** | - | - | ? | ? | - | - | - | - | D & V |
| Xue et al. | 2001 | **-** | - | - | - | - | - | - | - | - | D & V |

Legend: - indicates low concern; ? indicates unclear concern; + indicates high concern; D indicated development; V indicated validation.


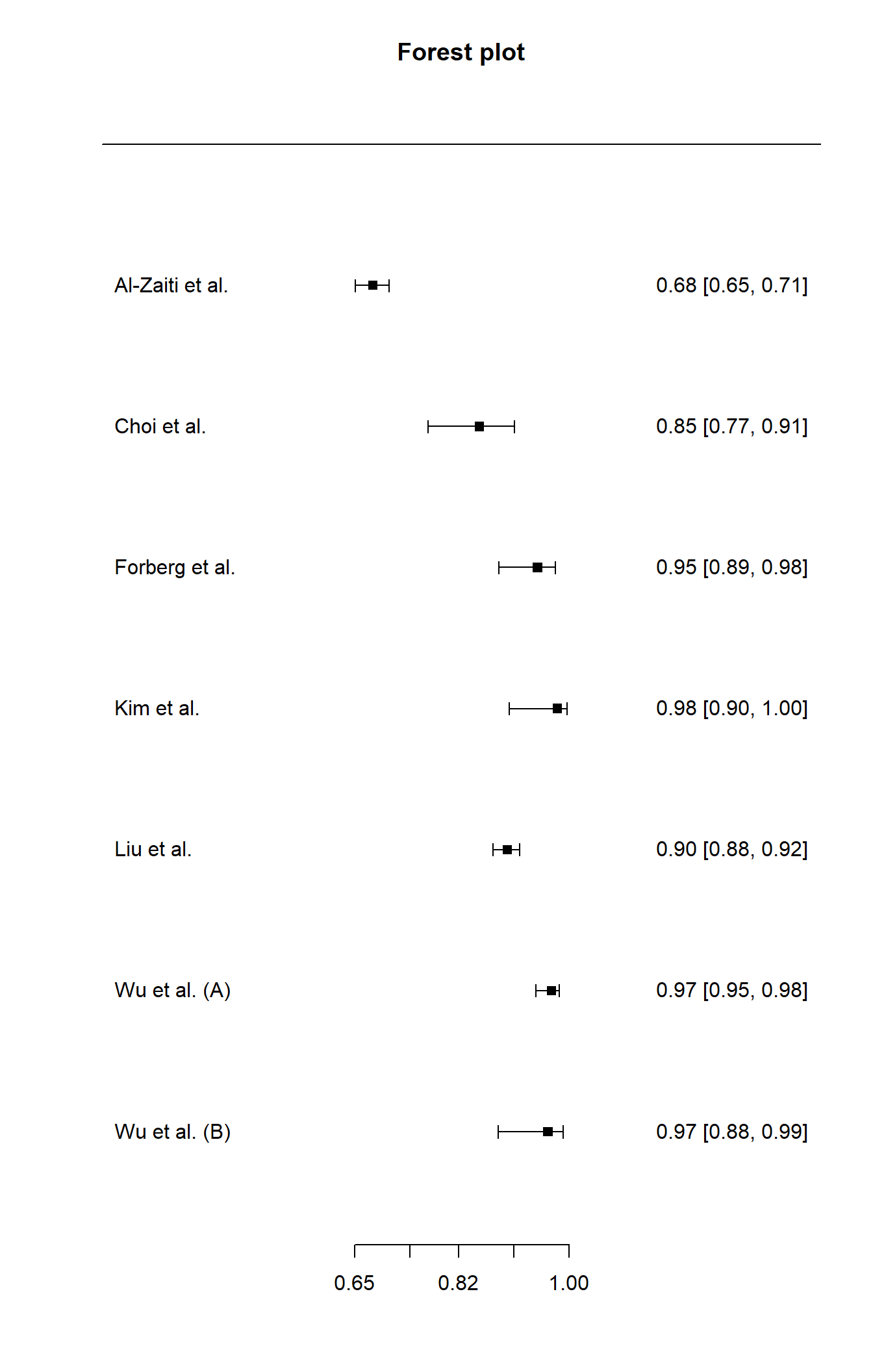


**Figure S1:** Forest plot of sensitivity of studies evaluating ST elevation myocardial infarction (STEMI) or occlusion myocardial infarction (OMI). Pooled sensitivity 92.5% (95% CI: 84.1%-96.6%).


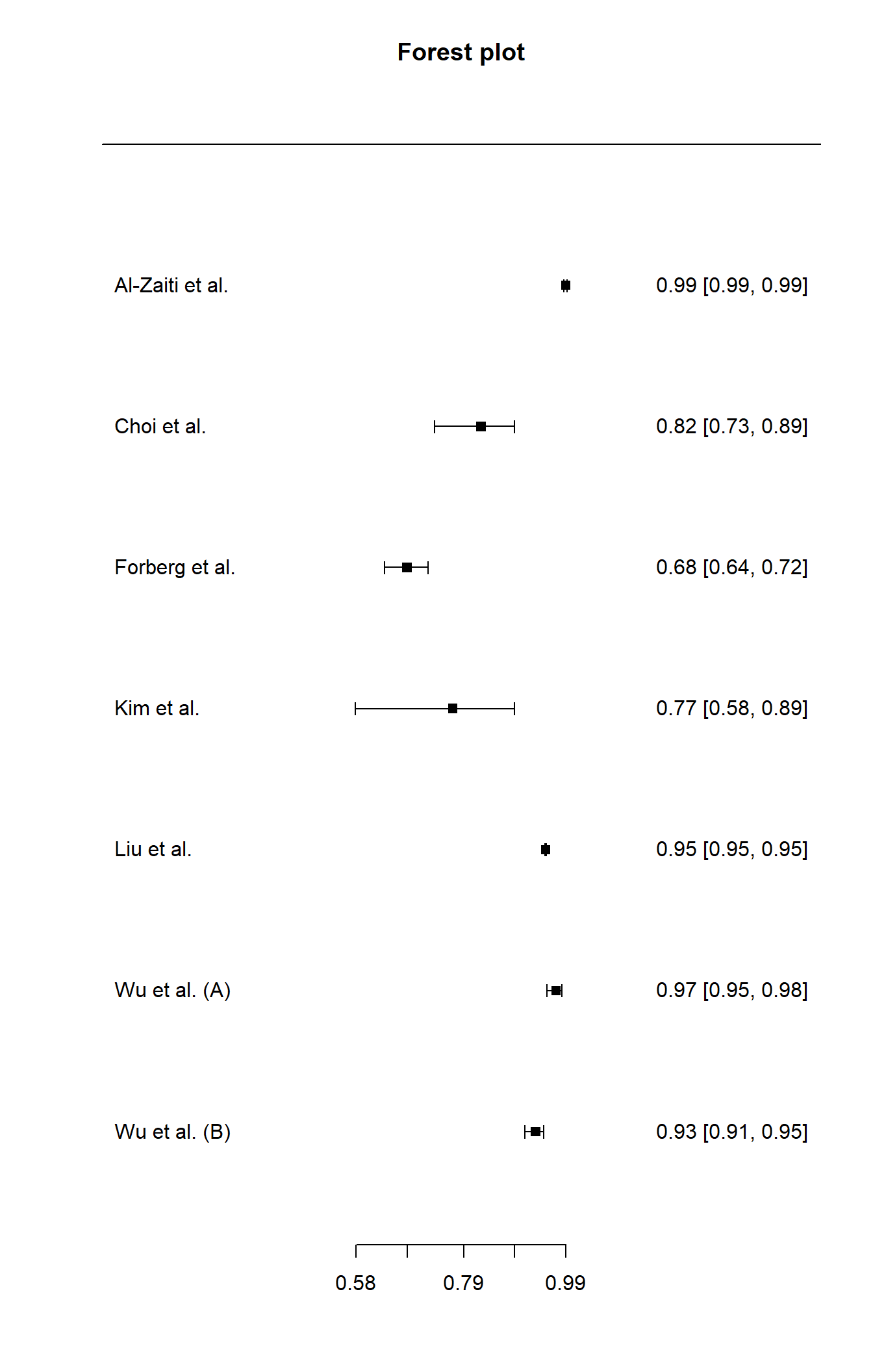


**Figure S2:** Forest plot of specificity of studies evaluating ST elevation myocardial infarction (STEMI) or occlusion myocardial infarction (OMI). Pooled specificity 92.0% (95% CI: 81.0%-97.0%).


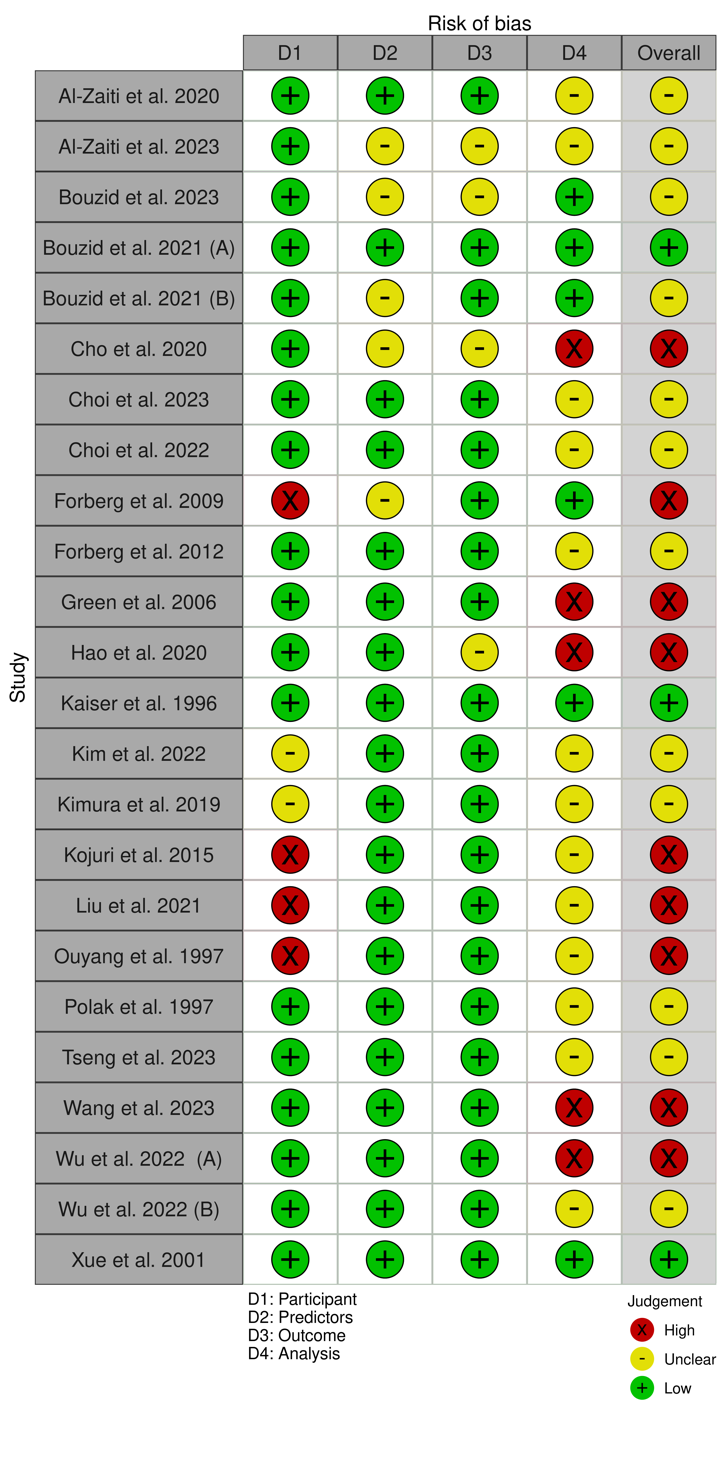


A


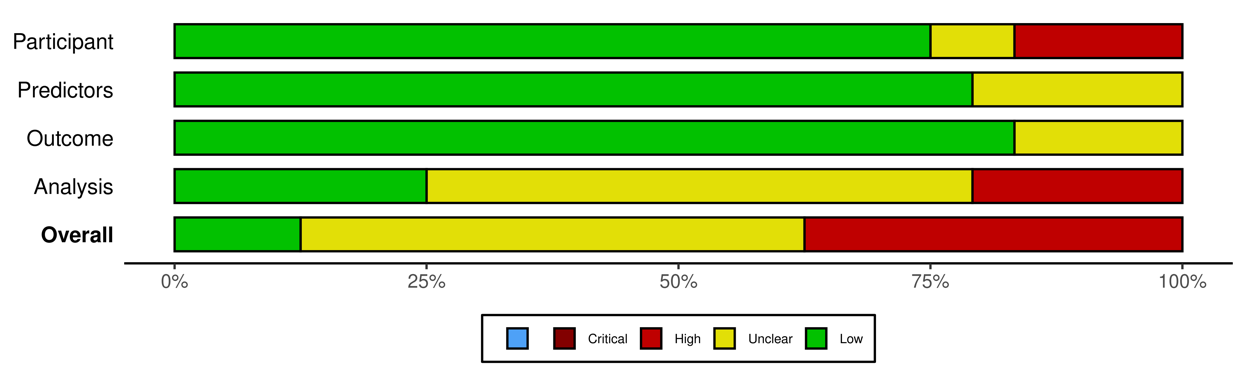


B

**Figure S3:** Risk of bias assessment using PROBAST analysis. Traffic plot (A) and summary (B).


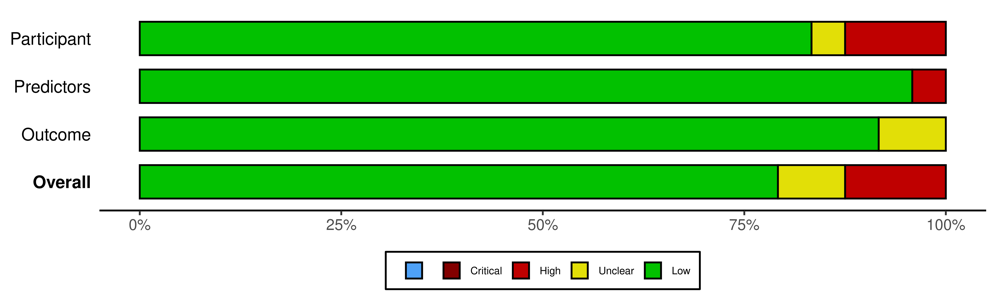

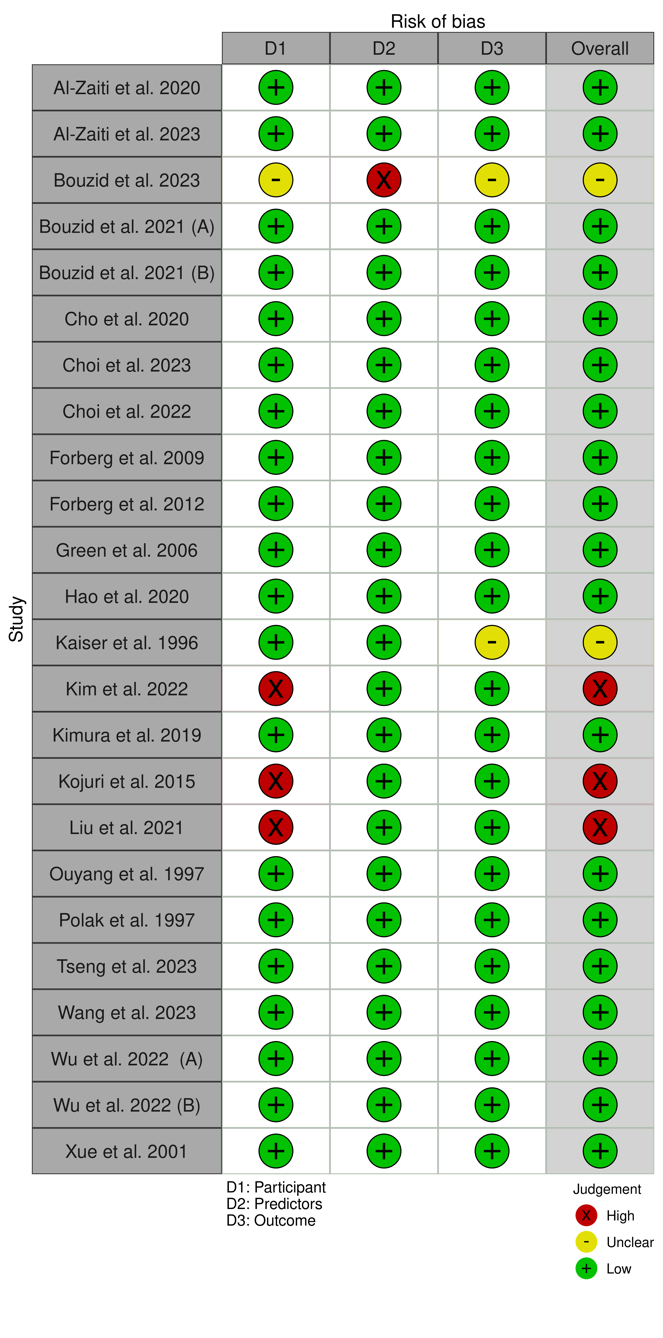


B

**Figure S4:** Applicability assessment using PROBAST analysis. Traffic plot (A) and summary (B).

**Supplementary figure 2:** Applicability assessment using PROBAST analysis. Traffic plot (A) and summary (B).

A
